# Spinal cord injury in severely injured patients: results from the Swiss Trauma Registry

**DOI:** 10.1186/s13049-025-01420-4

**Published:** 2025-06-05

**Authors:** Nader Hejrati, Felix C. Stengel, Michael G. Fehlings, Christian Maschmann, Martin N. Stienen, Kai O. Jensen, the Swiss Trauma Registry

**Affiliations:** 1 Department of Neurosurgery, Cantonal Hospital St. Gallen, HOCH Health Ostschweiz & University of St. Gallen, St. Gallen, Switzerland; 2 Spine Center, Cantonal Hospital St. Gallen, HOCH Health Ostschweiz & University of St. Gallen, St. Gallen, Switzerland; 3 Institute of Medical Sciences, University of Toronto, Toronto, ON M5S 1A8 Canada; 4 Division of Neurosurgery and Spine Program, Department of Surgery, University of Toronto, Toronto, ON M5T 1P5 Canada; 5 Emergency Department NFZ, HOCH Health Ostschweiz, Health & University of St. Gallen, St. Gallen, Switzerland; 6 EMS Regio144, Rüti (ZH), Switzerland; 7 Department of Traumatology, University Hospital Zurich, Zurich, Switzerland; 8 Swiss Trauma Board, Zurich, Switzerland

## Abstract

**Background and objectives:**

Traumatic spinal cord injuries (SCIs) in the context of severe trauma are rare, and patient demographics are infrequently reported. This study aimed to assess patient demographics in acute traumatic SCI in the context of severe injuries in Switzerland and to evaluate differences in demographics and outcomes stratified by timing of surgery.

**Methods:**

We analyzed data from the Swiss Trauma Registry (STR) from 2015 to 2024. The STR includes patients with major trauma (injury severity score [ISS] ≥ 16 and/or abbreviated injury scale [AIS] head ≥ 3) admitted to any level-one trauma centre in Switzerland. We evaluated patient characteristics, complications, and hospital outcomes, which were further stratified by early (< 24 h) and late (≥ 24 h) surgery.

**Results:**

Among 24,328 patients, 6,819 (28%) sustained spinal injuries, and 383 (1.6%) had a concurrent SCI with an incidence of 0.44 cases per 100’000 inhabitants. The median age was 52 years (IQR 31–70) and 73.6% were male. The primary causes were falls (63.1%) and road traffic accidents (29.6%). The in-hospital mortality rate was 4.7%. Late surgery patients more often had concomitant moderate or severe traumatic brain injuries (31% vs. 14%, *p* = 0.009) and were more likely to have no fractures or dislocations of the spine (22.8% versus 6.8%, *p* = 0.001). Patients who underwent early surgery had shorter hospital stays (9d [5-16], versus 16 d [9-24]; F = 13.92, *p* < 0.001). Late surgery was associated with a higher likelihood of developing two and more complications (OR 2.57, 95% CI 1.18–5.63, *p* = 0.018), including urinary tract infections (OR 12.13, 95% CI 2.76–53.41, *p* = 0.001) and multiple organ failure (OR 12.99, 95% CI 1.64-102.83, *p* = 0.015).

**Conclusions:**

This study offers insights into the characteristics and outcomes of acute SCI care in severely injured patients. Despite its low incidence, the acute management of this patient population remains highly challenging. Our findings suggest early stabilization of spinal injuries in severely injured patients may reduce hospital stays and complications.

## Introduction

Traumatic spinal cord injuries (SCIs) remain a significant global health challenge, often resulting in chronic impairment and disability for affected individuals [1]. Recent trends highlight an increasing prevalence of cervical incomplete SCIs in the elderly patient population, often occurring following minor trauma [2, 3]. Severely injured patients with multiple associated injuries constitute a particularly vulnerable subgroup within the traumatic SCI population. Up to 80% of patients sustaining traumatic SCIs from high-energy trauma, such as road traffic accidents or falls, present with multiple injuries, necessitating comprehensive and meticulous multidisciplinary care. Despite the profound and long-lasting impact of SCI on health-related quality of life and health care costs, epidemiological data on traumatic SCI in severely injured patients is sparse [4, 5].

Treatment of traumatic SCI is further challenged by the lack of effective neuroregenerative therapies to restore lost neurological function [6, 7]. Consequently, contemporary clinical management focuses on mitigating secondary damage through neuroprotective strategies [8]. A growing body of evidence supporting the benefits of early restoration of spinal cord perfusion through surgical decompression within 24 h of injury has reinforced the 2017 AO Spine recommendations on the timing of surgical decompression [9]. As a result, the updated 2023 AO Spine/Praxis Spinal Cord Institute guideline now strongly recommend offering early surgery within 24 h as an option for adult patients, regardless of injury level [10]. However, barriers to guideline implementation are present, particularly in low and middle-income countries (LMICs) where significant infrastructural disparities exist. A recent global AO spine survey revealed that logistic or administrative barriers to performing early surgery occur between 48% in high-income countries and 72% of cases in LMICs [11]. Given the vulnerability of severely injured SCI patients with multiple concomitant injuries, who require prioritized treatment of other systems according to the Advance Trauma Life Support protocol (ATLS) [12], surgical delays may be more likely in this group. The complex management of adult traumatic SCI in polytrauma has recently been addressed by the European Association of Neurological Surgeons (EANS) and the World Society of Emergency Surgery (WSES), who published a consensus and clinical recommendations paper on the topic [13]. However, a significant gap in high-quality evidence to support these clinical recommendations remains acknowledged.

The objective of this study was to assess patient demographics in acute traumatic SCI in the context of severe injuries from 2015 to 2024. Furthermore, our secondary objective was to evaluate differences in demographics and outcomes stratified by timing of surgery.

## Materials and methods

### Data source, study cohort

Data was derived from the Swiss Trauma Registry (STR; https://www.swisstraumaboard.ch). The STR was established in 2015 as a multi-center database to enable the standardized and pseudonymized documentation of patients with severe injuries. Severe injuries are defined as an injury severity score (ISS) ≥ 16 and/or an abbreviated injury scale (AIS) head score ≥ 3 [14]. Data are collected prospectively across different time phases, including pre-hospital care, emergency room and initial surgery, intensive care unit, and discharge.

Currently, twelve hospitals participate in the STR, submitting pseudonymized data to a central database via a web-based platform provided by Adjumed (https://www.adjumed.net). Participation in the registry is mandatory for these twelve trauma centers as part of quality assurance measures.

The STR database was accessed and queried on October 31, 2024. Data was derived from the STR for the period between January 01, 2015 and October 31, 2024 for all adult patients (≥ 16 years). For this analysis, only patients with a confirmed diagnosis of a SCI were included. This study has been assigned the project identifier STR-15.

### Ethical considerations

In accordance with Swiss law, the trauma registry is authorized under the Human Research Act (HRA). Our study was approved by the Regional Institutional Review Board of Eastern Switzerland (ID 2024 − 01325). Given the observational non-interventional nature of the study, which is based on a multicentric anonymized registry, informed consent was waved. None of the authors had access to information that could identify individual participants after data retrieval.

### Descriptors and characteristics

The following descriptors were considered: demographics (age, sex), trauma characteristics (date and time of trauma, mechanism of trauma, type of injury), Glasgow coma scale (GCS) at scene of accident, concomitant injuries, injury severity score (ISS), need for intensive care unit (ICU) care, duration of ICU stay in hours, length of hospital stay in days and discharge disposition. SCI related variables included the level of injury (cervical, thoracic, lumbar), injury severity (complete vs. incomplete) as well as the underlying spinal injury pattern (fracture, dislocation, fracture dislocation, no fracture or dislocation, presence of laceration). Additionally, surgical parameters included the timing of the index spinal surgery. The timing of surgery was calculated as the interval between the date and time of trauma and the date and time of the index spinal surgery.

### Study outcomes

The primary objective was to analyze the epidemiology of SCI in severely injured patients using descriptive statistics, and to evaluate trends in trauma mechanisms and patient demographics over the observation period (2015–2024). The secondary objective was to analyze patient demographics and outcomes stratified by the timing of surgery. A 24-hour cut-off was selected based on the existing evidence and guideline recommendations regarding the optimal timing of surgery for acute traumatic SCI [10, 15].

### Statistical analysis

The annual incidence of SCIs with concomitant severe injuries (as defined by an ISS of ≥ 16) was calculated using Swiss national population statistics from 2015 to 2024 [16].

Continuous variables were not normally distributed per the Shapiro-Wilk test. Hence, they were summarized using median with 1st and 3rd quartiles and compared between groups using the Mann-Whitney U test. Categorical variables were summarized using frequency count with percentage and compared between groups with chi-square test or the Fisher exact test, as appropriate. Univariate and multivariate analyses were performed for the analysis of timing of surgery and hospital outcomes. For continuous variables that were not normally distributed, a log transformation was performed prior to analysis. Differences in hospital and ICU length of stay between early (< 24 h) and late (≥ 24 h) surgical procedures were analyzed using multivariate analysis of variance (MANOVA). Results were reported with F-statistics. Logistic regression models were used for binary variables. For the multivariate analysis, adjustments were made for possible confounders that had a p-value < 0.1 in the baseline characteristics. To assess multicollinearity between predictor variables, Variance Inflation Factor testing was performed. Adjusted R-squared values were calculated for the linear and pseudo-R-squared values for logistic regression models and considered in the interpretation of the results. Results from logistic regression models are presented as odds ratios (OR) with 95% confidence intervals. All tests were two-sided and the significance level was set to 0.05. Data pre-processing and statistical analyses were performed in Stata version 18.0 (StataCorp LLC).

## Results

Between January 1, 2015 and October 31 2024, a total of 24,328 patients were enrolled in the STR. Of these, 6,819 (28%) sustained spinal injuries, and 383 (5.6% of those with spinal injuries) had a SCI. Overall, a concomitant SCI was observed in 1.6% of all severely injured patients in Switzerland. One patient was excluded from the analysis due to substantial missing data, leaving a total of 382 patients for the final analysis.

### Patient characteristics

Table 1 summarizes the baseline characteristics of the study cohort. The median age was 52 years (interquartile range [IQR] 31–70) and the great majority of patients were male (73.6%). Approximately two-thirds of the patients sustained very severe injuries based on the ISS (ISS 25–75), while the remaining one third had severe injuries (ISS 16–24). Falls were the most common trauma mechanism (*n* = 241, 63.1%) with 96.6% (*n* = 369) of patients sustaining blunt injuries. The spinal cord was most commonly injured at the level of the cervical spine (*n* = 234, 61.3%) followed by the thoracic (*n* = 108, 28.3%) and lumbar spine (*n* = 40, 10.5%). Fracture-dislocation injuries (*n* = 164, 53.4%) and fractures (*n* = 95, 30.9%) were the most common underlying injury pattern leading to SCI while 26.4% of patients (*n* = 100) had more than one spinal region injured. Incomplete SCIs (American Spinal Injury Association [ASIA] Impairment Scale [AIS] B-D) were observed in 57.3% (*n* = 211) of patients. Lastly, 17.4% of patients had additional moderate and severe traumatic brain injuries (TBIs).

**Table 1:** Baseline characteristics

Variables	All (*N* = 382)
Age, in years [IQR]	52 [ 31-70]
Male	281 (73,6%)
Trauma mechanism	
Falls Road traffic accident Other	241 (63.1%) 113 (29.6%) 28 (7.3%)
Injury mechanism	
Blunt Penetrating	369 (96.6%) 13 (3.4%)
Level of injury	
Cervical Thoracic Lumbar	234 (61.3%) 108 (28.3%) 40 (10.5%)
Injury Pattern Fracture-Dislocation Fracture Dislocation No Fracture or Dislocation	*(n = 307)* 164 (53.4%) 95 (30.9%) 18 (5.9%) 30 (9.8%)
Spinal cord injury severity Incomplete Complete	*(n = 368)* 211 (57.3%) 157 (42.7%)
Cord laceration	
No Yes	304 (79.6%) 78 (20.4%)
Number of spinal regions involved	
1 2 3 4	282 (73.8%) 77 (20.2%) 20 (5.2%) 3 (0.8%)
Injury severity score (ISS) Severe (ISS 16–24) Very severe (ISS 25–75)	*(n = 374)* 138 (36.9%) 236 (63.1%)
Number of other body parts & organs involved	
None 1–2 3–5 ≥ 6	125 (32.7%) 142 (37.2%) 88 (23%) 27 (7.1%)
Glasgow coma scale (GCS) 13–15 9–12 ≤8	*(n = 316)* 261 (82.6%) 20 (6.3%) 35 (11.1%)

### Incidence of SCI in severe trauma

The calculated incidence of SCI in severe trauma was 0.44 cases per 100’000 between 2015 and 2024, classifying it as an overall rare event.

### Trauma mechanisms and SCI types

**Fig. 1:**
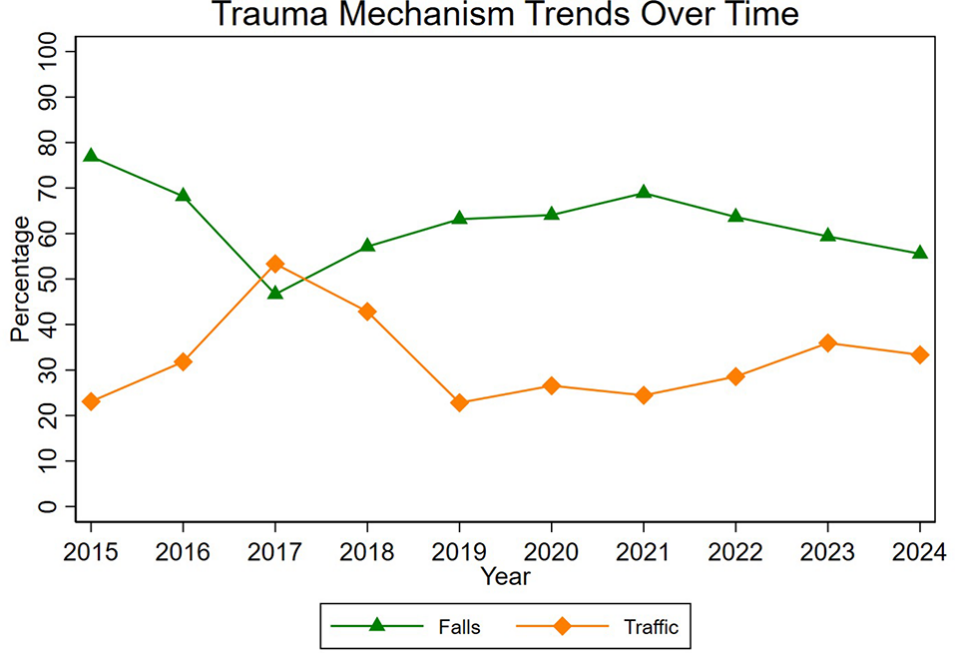
Trends of Trauma Mechanisms. Coefficient of Determination shows that trends of trauma mechanisms were not significant for both falls (R^2^ = 0.086, *p* = 0.411) and traffic accidents (R^2^ = 0.01, *p* = 0.779). The trends for the category of “other trauma mechanisms” is not depicted

**Fig. 2:**
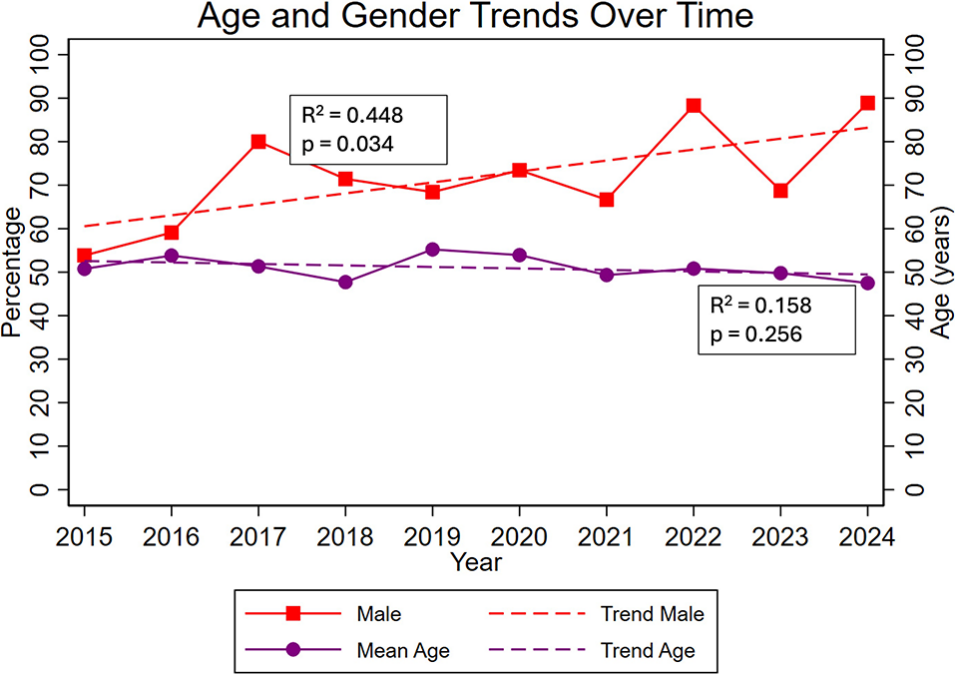
Trends of Age and Sex. Coefficient of Determination shows a significant positive trend for male sex (R^2^ = 0.448, *p* = 0.034). Trend of age was not signifcant (R^2^ = 0.158, *p* = 0.256)

Figure 1 highlights the trends underlying trauma mechanisms over the observation period. Overall, the distribution of trauma mechanisms remained relatively stable. There was an increasing trend in the proportion of male patients over years (R^2^ = 0.448, *p* = 0.034), while the average age showed a modest decline, which however, was not statistically significant (R^2^ = 0.158, *p* = 0.256; Fig. 2).

**Fig. 3:**
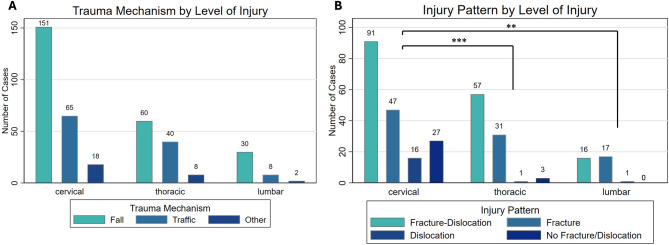
Trauma Mechanisms by Level of Injury. **A**. Falls were the most common trauma mechanism across all injury levels, followed by traffic accidents. **B**. Injury Pattern by Level of Injury. The most frequent injuries leading to SCI in the cervical and thoracic spine were facture-dislocations, followed by isolated fractures. Facture-dislocations and isolated fractures were nearly equally distributed in the lumbar spine. The cervical spine exhibited the highest number of spinal cord injuries caused by trauma without evidence of fractures or dislocations. Injury patterns were significantly different between cervical and thoracic (***p < 0.001) and cervical and lumbar SCIs (**p = 0.005)

As shown in Fig. 3A, falls were the most common trauma mechanism across all injury levels, followed by traffic accidents and other injury mechanisms. There were no significant differences in trauma mechanisms when compared by injury level. Regarding injury patterns, the most frequent injuries leading to SCI in the cervical and thoracic spine were facture-dislocations, followed by isolated fractures (Fig. 3B). In contrast, fracture-dislocations and isolated fractures were nearly equally distributed in the lumbar spine. Notably, the cervical spine exhibited the highest number of SCI’s caused by trauma without evidence of fractures or dislocations. As a result, the injury patterns were significantly different between cervical and thoracic (*p* < 0.001) and cervical and lumbar SCIs (*p* = 0.005).

### Timing of surgery for SCI

Table 2 outlines patient characteristics stratified by the timing of surgery (*N* = 348). Most patients (78.4%, *n* = 273) underwent early surgery within 24 h of injury with a median time of 6 h from time of accident [IQR 4.2–8.3]), while 21.6% (*n* = 75) underwent surgery ≥ 24 h after the injury with a median time of 46.1 h [IQR 31.9–97.1). The early surgery group tended to be younger (49.8 [31–69] vs. 55.0 [34–71] years, *p* = 0.076). Patients undergoing early surgery were more likely to have severe spinal column injuries, including fracture-dislocation type injuries, and had significantly higher rates of sensorimotor complete SCIs, (56.2% vs. 38.6%, *p* = 0.001; and 47.5% vs. 31%, *p* = 0.015, respectively). In contrast, patients who underwent late surgery had significantly higher rates of moderate and severe TBI’s (31% vs. 14%, *p* = 0.009). Interestingly, the severity of injury (according to the ISS) and the number of other body parts and organs involved did not significantly differ between the two groups.

**Table 2:** Patient characteristics stratified by timing of surgery (*N* = 348)

Variables	Early Surgery (*n* = 273, 78.4%)	Late Surgery (*n* = 75, 21.6%)	*P* value
Timing of surgery in h [IQR]	6.0 [4.2–8.3]	46.1 [31.9–97.1]	
Age, in years [IQR]	49.8 [31–69]	55.0 [34–71]	0.076
Male	205 (75.1%)	53 (70.7%)	0.458
Trauma mechanism			
Falls Road traffic accident Other	172 (63.0%) 85 (31.1%) 16 (5.9%)	46 (61.3%) 19 (25.3%) 10 (13.3%)	0.091
Injury mechanism			
Blunt Penetrating	266 (97.4%) 7 (2.6%)	69 (92.0%) 6 (8%)	**0.042**
Level of injury			
Cervical Thoracic Lumbar	159 (58.2%) 79 (28.9%) 35 (12.8%)	51 (68.0%) 21 (28.0%) 3 (4.0%)	0.065
Injury Pattern	*n* = 219	*n* = 57	
Fracture-Dislocation Fracture Dislocation No Fracture or Dislocation	123 (56.2%) 72 (32.9%) 9 (4.1%) 15 (6.8%)	22 (38.6%) 16 (28.1%) 6 (10.5%) 13 (22.8%)	**0.001**
Spinal cord injury severity	(*n* = 263)	(*n* = 71)	
Incomplete Complete	138 (52.5%) 125 (47.5%)	49 (69.0%) 22 (31.0%)	**0.015**
Cord laceration			
No Yes	215 (78.8%) 58 (21.2%)	60 (80.0%) 15 (20.0%)	0.874
Number of spinal regions involved			
1 2 3 4	202 (74.0%) 57 (20.9%) 12 (4.4%) 2 (0.7%)	52 (69.3%) 15 (20%) 7 (9.3%) 1 (1.3%)	0.166
Injury severity score (ISS)	*n* = 267	*n* = 73	
Severe (ISS 16–24) Very severe (ISS 25–75)	91 (34.1%) 182 (66.7%)	29 (39.7%) 46 (61.3%)	0.412
Number of other body parts & organs involved			
None 1–2 3–5 ≥ 6	84 (30.8%) 107 (39.2%) 65 (23.8%) 17 (6.2%)	21 (28.0%) 24 (32.0%) 20 (26.7%) 10 (13.3%)	0.185
Glasgow coma scale (GCS)	*n* = 240	*n* = 58	
13–15 9–12 ≤8	205 (82%) 14 (5.6%) 21 (8.4%)	40 (69.0%) 5 (8.6%) 13 (22.4%)	**0.009**

Data are presented as median [interquartile range] or number (percentage)

Timing of surgery was categorized into early = < 24 h from injury and late = ≥ 24 h from injury

### Hospital outcomes and complications rates

Hospital outcomes and complication rates are outlined in Table 3. The median length of hospital stay for the entire cohort was 11 days (IQR 6–18 days) with a median ICU stay of 92 h (IQR 37–231). The overall mortality rate was 4.7% (*n* = 18). Discharge outcomes showed that most patients were transferred to rehabilitation facilities (*n* = 228, 59.7%) or to another hospital for continued care (*n* = 118, 30.9%). More than half of the patients (55.5%) experienced at least one complication, particularly pneumonia (*n* = 56, 14.7%) and wound infections (*n* = 30, 7.9%).

**Table 3:** Hospital outcomes and complication rates

Variables		All (*N* = 382)
**Hospital outcomes**		
LOS, in days	[IQR]	11 [6–18]
Death	Yes, n (%)	18 (4.7%)
ICU stay, in hours	[IQR]	92 [37–231
Discharge disposition	Rehabilitation, n (%)	228 (59.7%)
	Other Hospital, n (%)	118 (30.9%)
	Home, n (%)	14 (3.7%)
	Died, n (%)	18 (4.7%)
	Unknown, n (%)	4 (1.0%)
**Complications**		
Number of complications	0	170 (44.5%)
	1	128 (33.5%)
	2	54 (14.1%)
	3	18 (4.7%)
	4	7 (1.8%)
	5	5 (1.3%)
Type of Complication*	Pneumonia, n (%)	56 (14.7%)
	Wound infection, n (%)	30 (7.9%)
	Urinary tract infection, n (%)	17 (4.5%)
	ALI/ARDS, n (%)	16 (4.2%)
	Pulmonary embolism, n (%)	15 (3.9%)
	Renal failure, n (%)	15 (3.9%)
	Sepsis, n (%)	14 (3.7%)
	Decubitus, n (%)	13 (3.4%)
	Deep vein thrombosis, n (%)	9 (2.4%)
	Cardiac arrest, n (%)	7 (1.8%)
	Multiple organ failure, n (%)	6 (1.6%)
	Stroke, n (%)	6 (1.6%)
	Myocardial infarction, n (%)	1 (0.3%)
	Compartment syndrome, n (%)	1 (0.3%)
	Other thromboembolic complications, n (%)	4 (1%)
	Other non-thromboembolic complications, n (%)	132 (34.6%)

Data are presented as median [interquartile range] or number (percentage)

Abbreviations: ALI: Acute lung injury; ARDS: Adult respiratory distress syndrome

* some patients had multiple complications

Table 4 presents hospital outcomes and complication rates stratified by the timing of surgery (*N* = 348). Patients in the late surgery group experienced significant longer hospital and ICU stays compared to those who were operated within 24 h (16 days [IQR 9–24], versus 9 days [IQR 5–16], F = 24.4, *p* < 0.001; and 199 h [IQR 60–363] versus 76 h [IQR 33–179], F = 13.9, *p* < 0.001). After adjusting for confounding variables, the timing of surgery remained a significant predictor of hospital LOS (F = 13.02, *p* < 0.001).

**Table 4:** Hospital outcomes and complication rates stratified by timing of surgery (*N* = 348)

Variables		Early Surgery (*n* = 273, 78.4%)	Late Surgery (*n* = 75, 21.6%)	Univariate Analysis OR (95% CI), *p*-value, F-statistics	Multivariate Analysis¹ OR (95% CI), *p*-value, F-statistics
**Hospital outcomes**					
LOS, in days	[IQR]	9 [5–16]	16 [9–24]	F = 24.38, ***p*< 0.001**	F = 13.92, ***p* < 0.001**^2,3^
Death	Yes, n (%)	11 (4.0%)	6 (8.0%)	2.07 (0.74–5.80), *p* = 0.166	2.60 (0.66–10.22), *p* = 0.172
ICU stay, in hours	[IQR]	76 [33–179]	199 [60–363]	F = 8.48, ***p* = 0.004**	F = 0.80, *p* = 0.373^2,3^
Discharge disposition	Rehabilitation, n (%)	170 (62.3%)	43 (57.3%)	0.81 (0.48–1.37), *p* = 0.437	0.91 (0.46–1.79), *p* = 0.789
	Other Hospital, n (%)	86 (31.5%)	18 (24.0%)	0.69 (0.38–1.24), *p* = 0.210	0.54 (0.25–1.18), *p* = 0.125
	Home, n (%)	4 (1.5%)	8 (10.7%)	8.03 (2.35–27.46), ***p* = 0.001**	19.04 (2.21–164.30), ***p* = 0.007**
	Died, n (%)	11 (4.0%)	6 (8.0%)	2.07 (0.74–5.80), *p* = 0.166	2.38 (0.59–9.61), *p* = 0.223
	Unknown, n (%)	2 (0.7%)	0 (0%)		
**Complications**	Yes, n (%)	153 (56%)	44 (59%)	1.11 (0.66–1.87), *p* = 0.684	0.93 (0.45–1.92), *p* = 0.854
Number of complications	0	120 (44%)	31 (41.3%)	1.11 (0.66–1.86) *p* = 0.685^4^	2.57 (1.18–5.63), ***p* = 0.018**^4^
	1	106 (38.8%)	16 (21.3%)		
	2	31 (11.4%)	16 (21.3%)		
	3	11 (4.0%)	6 (8.0%)		
	4	3 (1.1%)	3 (4.0%)		
	5	2 (0.7%)	3 (4.0%)		
Type of Complication*	Pneumonia, n (%)	33 (12.1%)	17 (22.7%)	2.13 (1.11–4.09), ***p* = 0.023**	1.92 (0.77–4.81), *p* = 0.163
	Wound infection, n (%)	21 (7.7%)	7 (9.3%)	1.24 (0.50–3.03), *p* = 0.644	1.40 (0.41–4.81), *p* = 0.597
	Urinary tract infection, n (%)	7 (2.6%)	8 (10.7%)	4.54 (1.59–12.96), ***p* = 0.005**	12.13 (2.76–53.41), ***p* = 0.001**
	ALI/ARDS, n (%)	8 (2.9%)	4 (5.3%)	1.87 (0.55–6.37), *p* = 0.320	4.79 (0.78–29.51), *p* = 0.092
	Pulmonary embolism, n (%)	11 (4.0%)	3 (4.0%)	0.99 (0.27–3.65), *p* = 0.991	1.00 (0.19–5.19), *p* = 0.998
	Renal failure, n (%)	11 (4.0%)	3 (4%)	0.99 (0.27–3.65), *p* = 0.991	1.10 (0.18–6.89), *p* = 0.915
	Sepsis, n (%)	11 (4.0%)	3 (4%)	0.99 (0.27–3.65), *p* = 0.991	1.13 (0.20–6.24), *p* = 0.892
	Decubitus, n (%)	7 (2.6%)	4 (5.3%)	2.14 (0.61–7.52), *p* = 0.235	0.57 (0.060–5.50), *p* = 0.629
	Deep vein thrombosis, n (%)	4 (1.5%)	3 (4.0%)	2.80 (0.61–12.80), *p* = 0.184	10.09 (0.71–142.60), *p* = 0.087
	Cardiac arrest, n (%)	3 (1.1%)	3 (4.0%)	3.75 (0.74–18.97), *p* = 0.110	2.18 (0.18–26.35), *p* = 0.539
	Multiple organ failure, n (%)	2 (0.7%)	4 (5.3%)	7.63 (1.37–42.52), ***p* = 0.020**	12.99 (1.64-102.83), ***p* = 0.015**
	Stroke, n (%)	4 (1.5%)	2 (2.7%)	1.84 (0.33–10.26), *p* = 0.485	2.39 (0.32–17.73), *p* = 0.395
	Myocardial infarction, n (%)	1 (0.4%)	0 (0.0%)	NA	NA
	Compartment syndrome, n (%)	1 (0.4%)	0 (0.0%)	NA	NA
	Other thromboembolic complications, n (%)	2 (0.7%)	2 (2.7%)	3.71 (0.51–26.81), *p* = 0.193	4.29 (0.42–44.37), *p* = 0.221
	Other non-thromboembolic complications, n (%)	96 (35.2%)	30 (40%)	1.23 (0.73–2.08), *p* = 0.441	0.85 (0.42–1.70), *p* = 0.640

Data are presented as median [interquartile range] or number (percentage)

Timing of surgery was categorized into early: <24 h from injury and late: ≥24 h from injury

¹Adjusted for age, GCS, level of injury, spinal cord injury severity, injury pattern, and trauma mechanism ²Linear regression coefficient for log-transformed LOS and ICU stay

³ Multivariate Analysis Of Variance (MANOVA) with F-statistics

^4^Logistic regression analysis after dichotomization of the number of complications into < 2 and ≥ 2 complications

Abbreviations: ALI: Acute lung injury; ARDS: Adult respiratory distress syndrome; CI: Confidence interval; ICU: Intensive care unit; IQR: Interquartile range; LOS: Length of stay; OR: Odds ratio

* some patients had multiple complications

Regarding complications, patients who underwent surgery ≥ 24 h after injury had higher incidence of multiple complications (≥ 2) compared to those operated within 24 h, effects which were significant following multivariate analysis (OR 2.57, 95% CI 1.18–5.63, *p* = 0.018). Additionally, the odds of developing urinary tract infections (OR 12.13, 95% CI 2.76–53.41, *p* = 0.001) and multiple organ failure (OR 12.99, 95% CI 1.64-102.83, *p* = 0.015) were significantly higher in patients who underwent surgery after 24 h.

## Discussion

We assessed national demographics of SCI in the context of severe trauma in Switzerland from 2015 to 2024. Our study shows that 1.6% (*n* = 383) of all severely injured patients in Switzerland sustain concomitant SCIs, with an overall estimated incidence of 0.44 cases per 100’000 inhabitants. Over the past decade, there has been a significant increase in male patients who comprise 73.6% of our study population. Falls (63.1%) and road traffic accidents (29.6%) remain the two leading trauma mechanisms with their proportions remaining relatively stable over the past decade. The majority of SCI cases (67.3%) occur in patients with multiple injuries rather than as isolated spinal injuries. Additionally, one in four patients sustained injuries in more than one spinal region (i.e., cervical, thoracic, lumbar, or sacral) while the cervical spinal cord was the most commonly affected injury level (61.3%) followed by the thoracic spinal cord (28.3%).

Our findings on SCI rates in severely injured patients align with those of Burney et al., who reported 2.6% of patients in the United States Major Trauma Outcome Study (MTOS) were diagnosed with a SCI [4]. Despite limited research on this patient population since the early 1990s, interest in this topic has recently grown within the EANS and the WSES who established consensus-based recommendations [13]. Although the incidence remains low, the consequences of sustaining a SCI can be devastating, particularly in the setting of severe trauma, as the prioritization of other organ systems in accordance with the established ATLS protocols may inevitably lead to delays in targeted SCI care [12]. Currently available guidelines primarily focus on early surgical decompression within 24 h of injury and blood pressure augmentation, aiming for mean arterial pressure (MAP)-targeted management to optimize spinal cord perfusion to improve neurologic outcomes [10, 17]. However, in patients with associated polytrauma, timely decisions regarding early surgery or MAP-targeted therapy remain particularly challenging. This is due to competing priorities, such as the need to manage life-threatening hemorrhages or uncontrolled bleeding, where lower MAP values may need to be tolerated as part of damage control resuscitation [18]. Furthermore, the accurate recognition of SCI can be hindered by concurrent injuries that complicate neurologic assessment, such as altered consciousness due to TBI, intubation or injuries of the extremities. Our study demonstrates, that 17.4% of patients have associated moderate to severe TBI and 67.3% present with multisystem injuries. A better understanding of the demographics of SCI patients in the context of severe trauma will aid in identifying key factors that require special attention during acute management and help optimize resource allocation.

While the distribution of injury levels aligns with previous reports on SCI in polytrauma patients [4], underlying injury patterns significantly differed between cervical and thoracic SCIs (*p* < 0.001) and cervical and lumbar SCIs (*p* = 0.005). Notably, cervical SCIs were more frequently associated with neither fractures nor dislocation-type injuries. While this has not been reported before in the context of polytrauma, it is consistent with observations in the overall SCI population [19–21]. Age-related degenerative changes of the cervical spine resulting in spinal stenosis are becoming increasingly prevalent, which are known to predispose to SCIs in the setting of trauma. Although the mean age of our patient cohort did not significantly increase over the past decade, it remains higher than in previously published studies [2, 4, 9], which may be a reflection of the age demographics of Switzerland [16]. As a result of the ageing demographic, traumatic SCIs without evidence of spinal column injuries, have become a focus of ongoing research, as SCIs in the elderly population have emerged as a growing global health concern [3, 22].

Despite the challenges in acute care of severely injured patients with SCIs, the majority of patients (78.4%) still received surgery within 24 h of injury. This is in contrast with previous studies, which report early surgery rates within 24 h ranging between 12.1% and 59% in their cohorts [15, 21–24]. Only one study reports comparable early surgery rates of 85.4% in their cohort of 96 motor complete SCI patients [25]. The reasons for the high rate of early surgery in our cohort need to be determined. However, Switzerland’s nationwide network of twelve level I trauma centers ensures rapid access to acute medical care with short transport distances between the trauma scene and the treating hospital. This is further supported by a well-established helicopter medical service which can significantly shorten rescue times and improve outcomes of severely injured patients [26]. Nevertheless, achieving early surgery remains a global challenge, particularly in LMICs, where logistic barriers - such as limited economic resources, medical equipment, operating room access and trained personnel - pose significant challenges [11, 27, 28].

Late surgery was more common in patients with moderate to severe TBI. A recent study of 14’964 patients with surgically managed traumatic SCIs, including 4’610 with concomitant TBI, found that TBI was independently associated with surgical delays in traumatic SCI patients (OR 1.3, 95% CI 1.1–1.6). Whether these delays are solely due to prioritization of acute TBI care or partially result from missed diagnoses due to altered consciousness remains to be investigated.

Timely recognition and referral of patients to spine surgeons remain challenging, particularly in mild forms of SCI, or in patients without radiographic evidence of fractures or dislocations [21]. Our findings indicate that patients who underwent late surgery were more likely to have incomplete SCIs and more likely to show no fractures or dislocations on initial polytrauma CT, despite similar ISS and number of affected systems across groups. This raises concerns about delayed diagnosis in patients with mild SCI and no signs of injury on imaging, a challenge further exacerbated by the complexities of managing multisystem trauma. These findings underscore the need for stronger evidence to educate clinicians and facilitate effective knowledge transfer regarding the role and timing of surgery. Given the importance of adequately assessing neurological impairment and the limitations of the ASIA scoring system in mild SCI, the ongoing AO Spine-sponsored IN-TWIN-study (Traumatic Incomplete Tetraplegia without Instability, NCT 05653206) aims to evaluate the feasibility of implementing additional and complementary outcome measures for cervical SCI patients with incomplete tetraplegia and no spinal instability [29].

The overall acute care hospital mortality rate in our study population was 4.8%, aligning with previous studies that report mortality rates ranging from 2 to 6.6% [2, 30]. Interestingly, Burney et al., whose study cohort also included SCI patients in the setting of severe trauma, reported a significantly higher mortality rate of 17% [4]. The substantially lower mortality in our cohort may reflect continued advancements in prehospital care and trauma center management for patients with acute SCI over the past 30 years.

Overall, complications occurred in 55.5% of patients, with pneumonia being the most common (14.7%). When further stratified by timing of surgery and adjusted for confounding variables, patients in the late surgery group had significantly higher rates of two and more complications and significantly higher rates of UTIs and multiorgan failure. Previous studies examining the impact of timing of early vs. late surgery using a 24-hour threshold have reported complications inconsistently and often without clear specification [15]. However, there is general consensus that early surgical stabilization improves non-neurological outcomes, such as hospital LOS, ICU duration, and rates of pneumonia and other complications, likely by promoting early mobilization and reducing immobility-associated risks [5, 31–33]. While establishing causality is challenging due to the observational nature of our analysis, our findings align with previous studies demonstrating that early surgery is associated with significantly shorter hospital stay, reinforcing the role of early surgery to improve non-neurological outcomes.

### Strengths and limitations

The strength of the study lies in its minimal missing data burden. In accordance with Swiss law, the STR has been mandatory since January 1st, 2015 across all twelve hospitals authorized to treat patients with severe injuries. This comprehensive nationwide implementation enables an accurate representation of the demographic data for the SCI population among severely injured patients.

Due to the observational nature of this study, establishing causal relationships remains challenging because of the inherent limitations of a non-randomized study design and the potential of introducing a systematic bias for more severe injuries to undergo late surgery. On the other hand, the registry-based approach allows for the inclusion of patients in a consecutive manner and, therefore, more closely reflects real-world conditions whereas an RCT may need to rely on narrow inclusion criteria and a controlled setting. Furthermore, patients with isolated SCIs, such as cord contusions without associated spinal column damage, may not meet the inclusion criteria of the STR. As a result, the dataset captures individuals with SCIs occurring in the context of severe trauma, defined by an ISS ≥ 16 and/or an AIS head score ≥ 3, and therefore does not represent the full spectrum of SCI cases. This focus on severely injured patients may partially account for the rapid assessment and treatment observed in the cohort. Finally, we were unable to address specific questions regarding neurologic outcomes in this study because the data source provided no data in this regard. Given that a large proportion of patients with SCIs are referred to one of the four nationwide paraplegia rehabilitation centers, future research could combine data from the STR with outcome registries such as the European Multicenter Study about Spinal Cord Injury (EMSCI) to explore the impact of acute SCI care on long-term neurological outcomes.

## Conclusion

This study offers a clearer insight into the characteristics and outcomes of acute hospital care for SCI patients with severe trauma treated at trauma centers across Switzerland. Between 2015 and 2024 the incidence of acute traumatic SCI among severely injured patients remained low. However, despite its relatively low incidence, the acute management of this patient population remains highly challenging. Our findings further support the notion that early stabilization of spinal injuries in trauma patients may reduce overall hospital stay while reducing the rates of complications. With a deeper understanding of the demographics of SCI patients in the context of severe trauma, specific strategies for optimal resource allocation can be developed and evaluated.

## Data Availability

The data that support the findings of this study are not openly available due to reasons of sensitivity and are available from the corresponding author upon reasonable request. Data are located in controlled access data storage at the HOCH Health Ostschweiz Cantonal Hospital St. Gallen.

## Abbreviations

AIS: Abbreviated Injury Scale
ASIA: American Spinal Injury Asosciation
ATLS: Advanced Trauma Life Support
CT: Computed Tomography
EANS: European Association of Neurological Surgeons
GCS: Glasgow Coma Scale
HRA: Human Research Act
ICU: Intensive Care Unit
ISS: Injury Severity Score
LMIC: Low and Middle- Income Country
LOS: Length of stay
MANOVA: Multivariate analysis of variance
MAP: Mean arterial pressure
MTOS: Major Trauma Outcome Study
RCT: Randomized controlled trial
SCI: Spinal cord injury
STR: Swiss Trauma Registry
TBI: Traumatic brain injury
UTI: Urinary tract infection
WSES: World Society of Emergency Medicine
